# *Listeria monocytogenes* meningitis

**DOI:** 10.1002/ccr3.1419

**Published:** 2018-04-17

**Authors:** Takahiro Matsuo, Nobuyoshi Mori, Aki Sakurai, Keiichi Furukawa

**Affiliations:** ^1^ Department of Infectious Diseases St. Luke's International Hospital Tokyo Japan

**Keywords:** Gram‐positive bacillus, *Listeria monocytogenes*, meningitis, tumbling motility

## Abstract

Tumbling motility is one of the useful characteristics of *Listeria monocytogenes*. This can be helpful to identify the causative pathogen along with Gram staining before the confirmatory microbiological examination.

A 79‐year‐old woman presented with high fever and headache for 2 days. On admission, cerebral spinal fluid (CSF) analysis revealed pleocytosis with polynuclear leukocyte predominance. CSF cultures revealed blue–gray colonies that comprised gram‐positive bacilli with tumbling motility (Video S1), which was turned out to be *Listeria monocytogenes*. Ampicillin and gentamicin for 3 weeks were administered and eventually, her symptoms improved. *Listeria* is an aerobic, beta‐hemolytic, gram‐positive bacillus, which exhibits tumbling motility on light microscopy 1. This characteristic of *L. monocytogenes* is evident through peritrichous flagella movement and is clinically useful to corroborate high suspicion of *Listeria* infection before the confirmatory microbiological examination.

## Conflict of Interest

Nothing to declare.

## Authorship

NM: assisted in the preparation of the manuscript. AS: made critical review of the manuscript. KF: approved the article finally.

## Supporting information


**Video S1.** “Tumbling motility” of Listeria monocytogenes.
